# Mr. Bishop, on Suspended Animation

**Published:** 1801-10

**Authors:** T. Bishop

**Affiliations:** Member of the Royal College of Surgeons, London. Leicester


### *To the Editors of the Medical and Phyſical Journal.*

Gentlemen,

The following relation of a caſe of ſuſpended animation which fell under my own immediate cognizance, being ſomewhat remarkable for the ſubject of it recovering ſufficiently to expreſs his ideas in very intelligible language, yet ſurviving only about 21 hours, I ſend you for inſertion in your miſcellany, if you deem it worthy of so much notice. It will be requisite to premise, that I was the active instrument of this person's partial recovery when a young apprentice to Mr. Watts, now Dr. Watts, a very superior and scientific man, of Cranbrook, in the western part of Kent, who did not see the patient till respiration had been established. The subjoined remarks upon this topic in general, you will retrench wholly, or in part, or not, as you may think proper.

I am, &c.

*Leicester, July* 30, 1807.

T. BISHOP,
Member of the Royal College of Surgeons, London.

---

John Morfatt, a butcher, of Cranbrook, a muscular, robust man, aged about 29 years, in a state of melancholia, attempted in the autumn of 1792, to terminate his existence by suspending himself to a beam in his slaughter-house; and so far accomplished his object, that he was not discovered till he had hung thus nearly ten minutes, when the cord was cut, and he was suffered to fall precipitately to the floor, which was paved, a distance of about fourteen feet. About fifteen minutes afterwards, the vital functions being completely suspended, the countenance of a deep purpule hue, I made a large orifice in one of the veins of the arm, with a view of taking away 6 or 7 ounces of blood; but an old, very ignorant surgeon arriving at this moment, compelled me to take away at least a pound of blood, which was done, and thus another quarter of an hour was expended. Notwithstanding no tremor of the heart, or subordinate vessels, or any other appearance of action could be discerned (excepting this ready flow of blood from the wounded vein, which I apprehended rather was independent of any muscular contraction in the vessels), this person, supposing the case should now be conducted like an ordinary fainting fit, directed me to have the body placed out of doors, to have a free admission of air, and to apply volatile alkali to the nostrils. But the body losing its heat rapidly, he modestly gave up the case. I then, i. e. at least 44 minutes after respiration had ceased, placed the body in a warm bed, and, being unacquainted with the practice of inflating the lungs, as speedily as possible threw into the rectum the smoke of burning tobacco very freely for the space of a quarter of an hour, heat being applied at the same time to the surface of the body by ordinary means. Two or three grains of tartarised antimony in solution were now put into the fauces; which producing no sensible effect, a spoonful or two of brandy and water was also made use of. Some time after after the above treatment had been adopted, i. e. about 15 or 20 minutes after he had been put to bed, and at leaſt 55 minutes after reſpiration had ceaſed, the colour of his lips was obſerved to become more florid, and now a ſlight degree of tremulous motion of the cheſt took place, which quickly amounted to a very laborious and imperfect reſpiration, accompanied with very ſlight convulſive contractions of the whole muſcular frame; but which, the reſpiration becoming ſtronger and ſomewhat more perfect, abated; and he ſoon afterwards appeared to be ſenſible of pain, which was expreſſed by groaning. In about an hour after the recommencement of reſpiration, this function became very much impeded by an abundant effuſion of mucus thrown out into the air-tube: he looked around him in great apparent diſtreſs of body and mind; the convulſions now greatly increaſed, affecting ſtill the whole frame, and betraying the epileptic character, in recuring by paroxyſms, each of which continued 10 or 15 minutes, till being exhauſted he fell into a ſtate of profound coma; and thus remaining from a quarter to half an hour, he arouſed and relapſed into another convulſive fit; each ſucceeding one abating of its violence, in proportion to his exhauſtion, for the ſpace of 18 hours. The impediment to his reſpiration, during the convulſive ſtruggles, which were at firſt equal to the coercive endeavours of four ſtout men, was extremely diſtreſſing to the ſpectator, reſembling a good deal the bellowing of a young calf. Fifteen hours after he had began to breathe, he was aſked if he recollected any perſon preſent: on which, looking around more collectedly than he had done before, appearing to be in great agony, he noticed, by a conſcious glance, his wife in particular, replying in the affirmative. He was aſked, how he felt himſelf? "Very bad."—If he wanted any thing?—"For God's ſake let me get to the window for air." He now relapſed into another fit, reſpiration became more free; the pulſe, which had hitherto been very irregular, now ſunk: having lain about two hours in a ſtate of inſenſibility, he expired quietly, within 21 hours after reſpiration had been reſtored.

If this caſe be worth thus recording in detail, it is chiefly ſo, becauſe it tends to ſhew in a ſtrong point of view, under what very diſadvantageous circumſtances the remaining irritability of the moving fibre may be arouſed, ſo as again to put into motion the great engine of life, even after its action had been ſuſpended nearly an hour, and this probably by means, which, from their compound qualities, proved ultimately deſtructive of the life they had ſo eſſentially conduced to reſtore. We were not permitted to examine the body after death, which might have developed the nature of the miſchief which was the remote remote caufe of death; but had it difcovered congeftion in the blood veffels of the brain, or even a breach in them and confequent effufion, it might have been queftioned whether fuch were the effects of the ligature around the neck, or of the deleterious properties of the tobacco. The convulfions were not remarkably violent on their firft appearance, but gradually increafed, and remitted as the refpiration became fomewhat eafy; raging again as it became more impeded, which happened in about an hour after he had begun to breathe. This, I apprehend, to be in fome degree equivocal evidence, that the convulfions and dyfpnœa were joint effects of the tobacco, which eventually exhaufted fo completely the living powers that they could rally no longer; or, why did not thofe fymptoms occur fooner, preffure on the brain ufually manifefting itfelf as foon as it is applied? On a fuppofition that the violence originally committed to the brain was the remote caufe of his death, it is curious that the brain under fuch circumftances fhould admit of fo perfect a reftoration of its own functions at firft; and more fo, that, when thus complete, it fhould yet not be able to fupport them a longer period.

The ufe of tobacco is now, I believe, univerfally difcontinued upon thefe occafions, in this country; M. Portal had long fince prohibited it in France. If any hazard attend its ufe in thefe cafes, it may be occafionally noxious in others. I have feen it, when one drachm of it had been employed in infufion for a glyfter, fo immediately deprefs the ftrength of an old man labouring under the milder fymptoms of a ftrangulated hernia, that he furvived but a very fhort period after its ufe, when fo fudden a change had not been indicated the moment before it had been employed.

It has frequently happened, in attempting to recover perfons apparently dead, in fainting fits and in articulo mortis, that fluid poured into the mouth has efcaped into the larynx; which muft often happen if all the parts concerned in deglutition do not act in concert with each other, and more particularly if they do not act at all, as well when the head is held obliquely, for in that pofition the epiglottis does not entirely cover the aperture of the glottis. No fluid therefore fhould be exhibited under fuch circumftances in fuch a manner; it can be given fafely only by means of a tube which reaches the upper orifice of the ftomach. There is no queftion about the fuperior utility of taking off the compreffion made upon the pulmonary artery when refpiration has ceafed in thefe cafes, and which alone has fo often fucceeded in reftoring it when it had not long ceafed: but this ftep, aided by the electric fhock, however properly conducted, would probably never fucceed

ſucceed when the blood has almoſt entirely parted with its heat, without an additional ſupply of that ſtimulus. When bellows, or other apparatus adapted to this purpoſe are not at hand, a ſufficient quantity of air may commonly be forced into the lungs by alternately depreſſing the ſternum and the cartilaginous extremities of the ribs, and ſuffering their elaſtic reaction to follow regularly. This mode I find to be completely adequate to the end propoſed in the younger ſubject, the flexibility of the ribs being ſo much greater than that in the adult: That the lungs are actually diſtended by this mode may be in ſome meaſure determined by the effect of the expired air on the flame of a candle. If any difficulty ariſe in thus inflating the lungs, and a convenient tube be at hand, it ſhould be put through the noſtrils into the larynx, in order to ſuperſede the neceſſity of making an artificial opening in that part of the air tube; but if this, which is not manageable without difficulty, do not ſucceed well, or cannot be tried, ſuch an opening ſhould be made. If any hæmorrhage enſue from the wound, whether veinous or arterial, the bleeding veſſel ſhould be immediately ſecured; for if blood get into the trachea, and the patient recover, it will at leaſt prove diſtreſſing. Such an occurrence had nearly deſtroyed a patient lately, on whom this operation had been employed by a very eminent ſurgeon and anatomiſt, on account of an enlargement of the tonſils, who ſaw no neceſſity to tie any veſſel while he remained with his patient, and reached him but juſt in time to ſave him by tying the veſſel.

From the impreſſion which the electric ſhock communicates to a body recently but irrecoverably dead, which I have witneſſed, I have the higheſt opinion, as every one muſt have, of its power in caſes of ſuſpended reſpiration; but I apprehend it is very poſſible to employ it to an extent in which it would be more likely to extinguiſh than to rouſe the living power to action. But few probably will be diſpoſed, in want of an electrical apparatus, to apply the Galvanic excitement to the nerves termed par vagum, the intercoſtal and the phrenic, in their courſe down the neck; the expoſure of which for that purpoſe requires the cautious and diſcriminating knife of the anatomiſt, in whoſe hands I doubt not of the eligibility of ſuch an expedient in a caſe of deſperation.

